# Risk of colorectal adenocarcinoma in men receiving androgen deprivation therapy for prostate cancer; a nationwide cohort study

**DOI:** 10.1007/s10552-023-01736-5

**Published:** 2023-06-21

**Authors:** Richard Shore, Ji Zhang, Weimin Ye, Pär Stattin, Mats Lindblad

**Affiliations:** 1 Department of Clinical Science, Intervention and Technology (CLINTEC), Karolinska Institutet, Stockholm, Sweden; 2 Function Perioperative Medicine and Intensive Care, Karolinska University Hospital, Stockholm, Sweden; 3 Department of Medical Epidemiology and Biostatistics (MEB), Karolinska Institutet, Stockholm, Sweden; 4 Department of Surgical Sciences, Uppsala University, Uppsala, Sweden; 5 Department of Upper Abdominal Diseases, Karolinska University Hospital, Stockholm, Sweden

**Keywords:** Colorectal neoplasms, Adenocarcinoma, Prostatic neoplasms, Gonadal steroid hormones, Androgen antagonists.

## Abstract

**Purpose:**

To assess whether androgens play a role in explaining the sex related differences in the incidence of colorectal cancer (CRC).

**Methods:**

A nationwide matched cohort study was conducted employing the Prostate Cancer data Base Sweden (PCBaSe) 4.0 during the study period 2006–2016. Prostate cancer (PC) patients receiving androgen deprivation therapy (ADT) were treated as exposed. Prostate cancer-free men from the general population were randomly selected and matched to the index case by birth year and county of residence, forming the unexposed group. All were followed until a diagnosis of CRC, death, emigration, or end of the study period. The risk of CRC among ADT exposed PC patients compared to unexposed cancer-free men was calculated using a flexible parametric survival model and expressed as hazard ratios (HRs) with 95% confidence intervals (CIs).

**Results:**

There was an increased risk of CRC among ADT exposed PC patients compared to unexposed cancer-free men (HR 1.27 [95% CI 1.15–1.41]), in particular an increased risk of adenocarcinoma of the colon (HR 1.33 [95% CI 1.17–1.51]) and more specifically an increased risk of adenocarcinoma of the distal colon (HR 1.53 [95% CI 1.26–1.85]). Examination of latency effects yielded significantly decreased HRs over time for CRC (p = 0.049 for trend).

**Conclusions:**

This population-based study found an increased risk of CRC among PC patients exposed to ADT, specifically adenocarcinoma of the distal colon, which indicates an increased association between ADT (PC + ADT) and CRC but not a positive dose-response trend questioning a true causal effect.

## Introduction

Colorectal cancer (CRC) is the third most common cancer in men, the second most common cancer in women and the second most common cause of cancer death in both sexes when analyzed separately, accounting for 1 in 10 cancer cases and deaths globally [1]. In the past decades the incidence of CRC has increased globally, most of which can be explained by a growing and aging population, however, the incidence would have increased withholding population and age dynamics due to changing age-specific incidence rates [2] mostly attributable to the influence of new dietary patterns, obesity, life-style factors and a lack of health infrastructure and early detection [3]. The odds of developing CRC is higher in men than in women [2], whereas the age-specific incidence rates during the past decades is similar for men and women [4]. However, in more recent times there is a global trend for men to have higher age-specific incidence rates than women [5].

The proximal colon originates from the embryological midgut whereas the distal colon and rectum originate from the hindgut. The two subsites differ partly with regard to mechanisms of carcinogenesis [6] and genetic architectures [7] but a distinct dichotomy has been challenged [8]. The incidence of CRC in the proximal colon is higher in women than in men and the reverse is true for the distal colon and rectum [9]. Other established risk factors for CRC include hereditary factors, abdominal radiation, lifestyle factors, obesity, metabolic syndrome, hyperinsulinemia, and diabetes. Protective factors include daily moderate physical activity and a diet high in whole grains and dietary fibers [10, 11]. The sexes also differ with regard to exposure to risk factors such as smoking [12], alcohol [13], diet [13] and obesity [14]. Regarding sex hormones, higher endogenous testosterone levels have been linked to both a lower risk of CRC in men [15] and a higher risk of CRC in postmenopausal women [16]. Experimental studies have indicated that androgens may have a protective effect against colorectal carcinogenesis, decreasing CRC risk by activating androgen receptors [17]. Furthermore, testosterone deficiency, for example induced by androgen deprivation therapy (ADT) for prostate cancer has been associated with the development of CRC [18], as well as conditions [19, 20] which are known risk factors for CRC such as diabetes and metabolic syndrome [21–23].

ADT is either surgical (bilateral orchiectomy) or chemical (gonadotropin-releasing hormone (GnRH) agonists or oral anti-androgens) and has long been a standard treatment for metastatic prostate cancer and is also part of the treatment of prostate cancer patients with locally advanced or high-risk disease as neoadjuvant and adjuvant ADT in conjunction with radiotherapy [24–26]. However, ADT is increasingly being used earlier in the course of the disease, such as in patients with biochemical relapse, thus potentially allowing for more prostate cancer patients to be exposed to ADT for a longer period of time.

The relationship between androgens and the risk of CRC needs to be further examined. We therefore hypothesized that androgens decrease the risk of CRC and consequently that ADT increases the risk in men. We further hypothesize that the risk of CRC is dependent on the sub-site of the colon analyzed since they differ in origin and the incidence of CRC in the distal colon and rectum is higher in men. To test these hypotheses, and since the aim of this study was not primarily related to the clinical setting of prostate cancer patients and their risk/benefit of undergoing ADT, but rather to assess whether androgens play a role in explaining the sex related differences in the incidence of CRC, we identified a nationwide cohort of men with prostate cancer who were exposed to different forms of ADT and compared them to a matched cohort of unexposed and cancer-free men.

## Methods

### Data sources

We used data from Prostate Cancer data Base Sweden (PCBaSe), created when the nationwide and comprehensive National Prostate Cancer Register (NPCR) [27] in Sweden was linked to a number of other national health-care registers and demographic databases including the Total Population Register, Patient Register and Cause of Death Register [28, 29]. PCBaSe contains information from the NPCR, which covers > 98% of all diagnosed cases of prostate cancer compared to the Swedish Cancer Register, became nationwide in 1998 and includes information such as: age at diagnosis, date of diagnosis and data on primary prostate cancer treatment [28, 30, 31]. In this study, we collected data from the latest installment, PCBaSe 4.0, which includes data from 1998 to 2016, and in which a comparison cohort of men without prostate cancer has been created by selecting five prostate cancer-free men in a randomized fashion from the Total Population Register, matched to each of the index cases by birth year (attained age) and county of residence [32]. Data on migration was obtained from the Total Population Register [33] whereas death dates were obtained from the Cause of Death Register [34] and marital status and educational level from The Longitudinal integration database for health insurance and labor market studies (LISA) [35]. Data on drug use was linked to PCBaSe 4.0 from the Prescribed Drug Register [36], which contains data on all prescribed drugs in Sweden since its inception on 1 July 2005.

### Study design

We conducted a matched cohort study employing the PCBaSe 4.0 database. Entry time in the study was set from 1 January 2006 (to allow for left truncation of the Prescribed Drug Register as is required in all time-to-event analysis due to the later start date on 1 July 2005) [32] to 31 December 2015. Prostate cancer patients receiving ADT were included in the cohort as exposed, while prostate cancer patients that did not receive ADT and their matched controls were excluded from the study. We used the comparison cohort of men without prostate cancer described above as the unexposed group. This choice of control group was based not on the risk of confounding alone, but also on avoiding misclassification of ADT exposure, and that confining the study population only to men diagnosed with PC would raise concerns on its external validity and relevance for the general population. Men were followed until a diagnosis of colorectal cancer, death, emigration, or end of the study period 31 December 2016, whichever occurred first. Ascertainment of cancer cases was obtained by linkage with the Swedish National Cancer Register in which more than 98% of cancer cases are morphologically verified [37].

### Study population

The source population included all Swedish men diagnosed with prostate cancer during 2006 through 2015 and their matched controls wherefrom the study population was selected consisting of all ADT-exposed prostate cancer patients and their corresponding unexposed cancer-free matched controls. The index date was the date of prostate cancer diagnosis for cases, and the controls were assigned the same index date as their matching case. Follow-up was started at the date of the first ADT prescription for prostate cancer cases and the same date for the corresponding controls. Prostate cancer cases (and their matched controls) with another cancer diagnosis (apart from non-melanoma skin cancer ICD-7: 191) at or prior to start of follow-up, a missing date of prostate cancer diagnosis, or a follow-up time of less than one year were excluded. Furthermore, the first year of follow-up for both cases and controls was excluded to limit the effects of surveillance bias, i.e., patients with a newly diagnosed prostate cancer could be more likely to have another cancer detected due to the diagnostic evaluation of their prostate cancer.

### Exposure definitions

All included prostate cancer cases were ADT exposed and their corresponding cancer-free matched controls were considered unexposed to ADT. ADT was administered in the form of anti-androgens (AA), gonadotropin-releasing hormone (GnRH) analogues (i.e., agonists), GnRH + flare protection (by concomitant use of anti-androgens during a restricted time, usually one month), orchiectomy and total androgen blockade (GnRH plus anti-androgens continuously). Some cases were initially placed on watchful waiting at diagnosis and put on ADT at a later date. We only had information on the first prescription of ADT administered to each case of prostate cancer. Cases were considered exposed from the date of first prescription of ADT and were assumed to remain exposed to the same type of ADT until the end of follow-up. To identify incident use of ADT in men who started ADT as opposed to men who had used it for a long period with an unknown start date (before the inception of the Prescribed Drug Register) we needed a wash-out period of six months. Hence, all prostate cancer patients diagnosed before 1 January 2006 (from 1998 to 2005) and their matched controls were excluded from the study.

### Outcome definitions

**Fig. 1:**
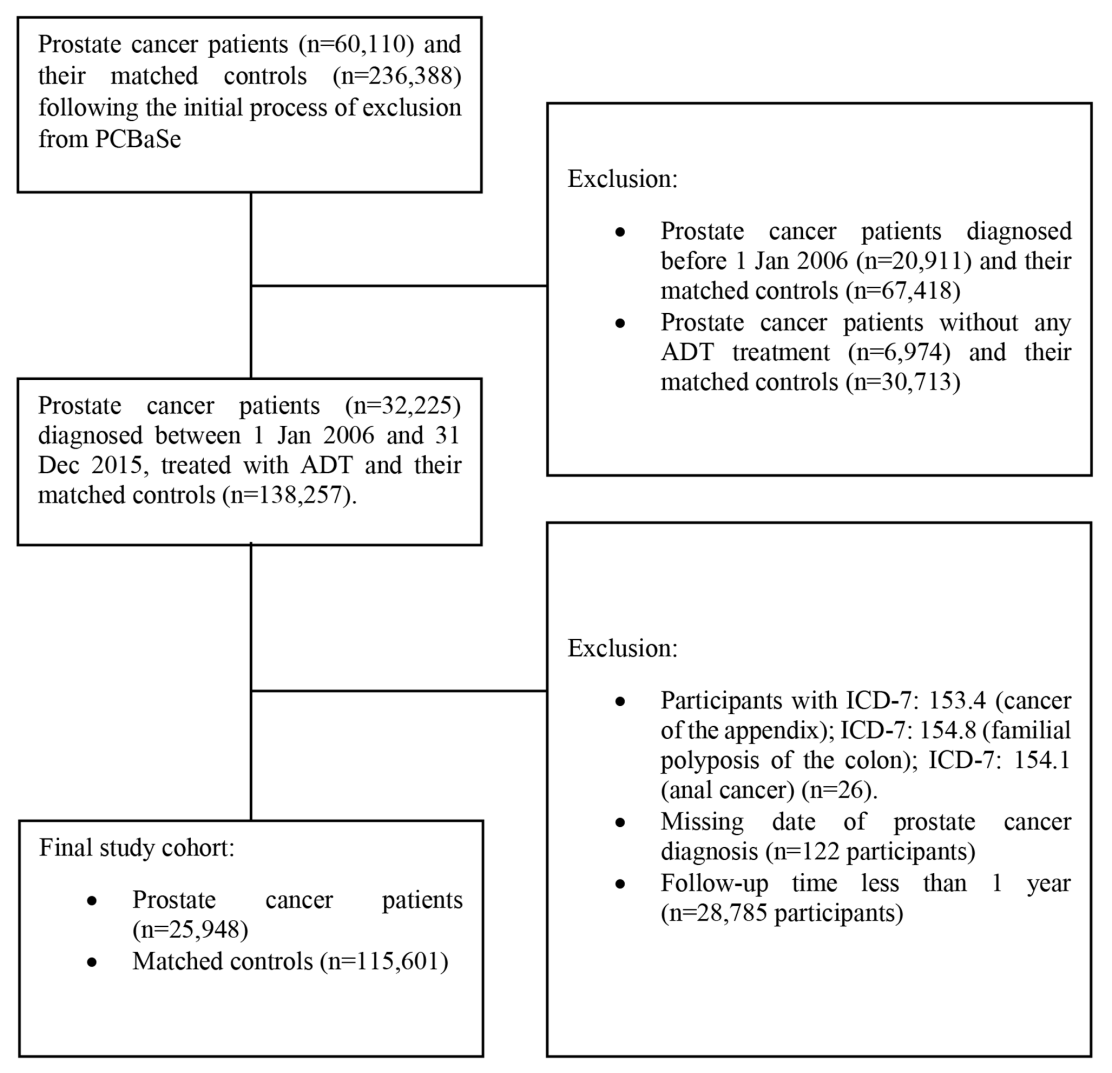
Flowchart of study cohort formation

Subtypes of CRC were classified using the 7th version of the International Classification of Diseases (ICD-7) and histology was classified according to WHO/HS/CANC/24.1 histology codes. For adenocarcinoma of the colon ICD-7: 153.0–153.3 was included. Cancer of the appendix (ICD-7 153.4) and Familial polyposis of the colon (ICD-7 153.8) were therefore not included as outcomes (Fig. 1) whereas overlapping lesion of the colon; colon unspecified including large intestine NOS (ICD-7 153.9) was included in the analysis of colorectal and colon cancer but not included as an outcome in the sub-site analyses of proximal and distal colon cancer. Among rectal cancers, cancer of the colorectal junction and rectum proper (ICD-7: 154.0) was included while not including anal cancer (ICD-7: 154.1) as an outcome (Fig. 1). The following WHO/HS/CANC/24.1 histology codes were used and jointly classified as adenocarcinoma: 093 (adenocarcinoma of unknown origin), 094 (adenocarcinoma in situ) and 096 (adenocarcinoma). Colorectal cancers with other histological classifications were omitted from the analyses. Firstly, we analyzed all included sub-sites of the colon and rectum combined. Secondly, we analyzed the colon and rectum separately. Thirdly, we separated the colon into the proximal and the distal parts. Finally, cases that were diagnosed with both colon cancer and rectal cancer simultaneously (double registration in the database) were included in the analysis of all sites of the colon and rectum combined but excluded from all other analyses.

### Statistical analyses

The continuous variables of the study population were summarized as means with standard deviations. The categorical variables were described using frequencies with percentages. Crude incidence rates with corresponding confidence intervals (CIs) of CRC in the ADT-exposed prostate cancer group and unexposed cancer-free control group were calculated by dividing the number of newly diagnosed CRC cases over accumulated person-time. The association between use of ADT, including subtypes, and the occurrence of different localizations of colorectal cancer was studied. Hazard Ratios (HRs) with corresponding 95% CIs for developing CRC were calculated comparing each ADT exposure group to the matching ADT-free control group, respectively. HRs were modeled by a flexible parametric survival model using follow-up time as the underlying timescale of research. We decided à priori to employ a flexible parametric survival model. For correlations within matching groups, all CIs were adjusted for the clustering effect. HR > 1 implied a higher risk for the outcome of interest, i.e., CRC or subsites of CRC. The model fitness of either time-constant or time-dependent were compared and since the results of the two different models did not differ substantially, suggesting no significant time-dependent effects, we decided to employ the time-constant model in all main analyses. We also controlled for the potential confounders of age (continuous), attained level of education (Low [< 10 years]; Middle [10–12 years]; High [> 12 years]) and marital status (not married, married, separated, widower). A complete case analysis was performed because the number of missing variables was considered negligible. To study the effect of latency in CRC development, we also grouped prostate cancer patients based on their exposure time to ADT into three separate groups: 1–3 years; 3–5 years; >5 years and modeled the HRs using a Poisson regression model. The HRs for developing CRC were calculated comparing each ADT exposure group to the matching ADT-free control group, respectively (non-cumulative). P-values < 0.05 were considered statistically significant. All statistical analyses were performed using the statistical software Stata (version 16; Stata Corporation, College Station, TX, USA). The study was approved by the Ethical Review Board in Stockholm, Sweden (DNR: 2009/1196-31/1 with amendments DNR: 2009/2124-32 and DNR: 2011/1674-32).

## Results

### Participants

Following initial exclusions, PCBase encompassed 60,110 patients with prostate cancer and 236,388 matched cancer-free controls. After exclusions (Fig. 1) 25,948 ADT exposed patients with prostate cancer diagnosed between 1 January 2006 and 31 December 2015 and 115,598 unexposed cancer-free controls remained for final analysis. The total follow-up time in the cohort was 521,226.0 person-years and the median follow-up time was 4.2 years (interquartile range 2.5 to 6.6 years). Characteristics of the study participants are presented in Table 1. There were no major differences between exposed and unexposed with regard to age, marital status, or educational level. The time from prostate cancer diagnosis to start of ADT ranged between 0 and 119 months: 65.3% of patients started ADT within 3 months of prostate cancer diagnosis; 14.9% of patients started ADT between 3 and 12 months following prostate cancer diagnosis; 19.8% of patients started ADT more than 12 months after prostate cancer diagnosis. The median (interquartile range 1–3) number of days of waiting time from prostate cancer diagnosis to start of ADT was 49 (25–210) days. The follow-up time was slightly longer for unexposed than ADT exposed.

**Table 1:** Characteristics of men exposed or not exposed to androgen deprivation therapy (ADT) in Prostate Cancer data Base Sweden

	Non-ADT exposed (n, %)	ADT exposed (n, %)
Participants	115,598 (81.7)	25,948 (18.3)
Mean age at start of follow-up (years)	74.6 ± 8.7	75.0 ± 8.6
Mean follow-up time (years)	4.8 ± 2.6	4.1 ± 2.4
Marital status		
Not married	12,687 (11.0)	2,658 (10.2)
Married or registered partnership	72,375 (62.6)	16,344 (63.0)
Separated (/registered partnership)	16,105 (13.9)	3,579 (13.8)
Widower (/registered partnership)	14,431 (12.5)	3,367 (13.0)
Education		
Low (< 10 years)	50,661 (43.8)	11,789 (45.4)
Middle (10–12 years)	41,138 (35.6)	9,155 (35.3)
High (> 12 years)	21,909 (19.0)	4,717 (18.2)
Missing values	1,890 (1.6)	287 (1.1)

Continuous variables expressed as mean ± standard deviation. Categorical variables expressed as frequencies (n) with percentages (%)

### Risk of colorectal adenocarcinoma

During follow-up, 2,243 participants in the cohort developed a new diagnosis of CRC. Exposure to any form of ADT among prostate cancer patients was followed by an increased risk of CRC (HR 1.27 [95% CI 1.15–1.41]) as compared to ADT unexposed cancer-free matched controls. Stratified into different forms of ADT, there was a significantly increased risk of developing CRC after exposure to AA (HR 1.23 [95% CI 1.04–1.47]) and GnRH + flare (HR 1.22 [95% CI 1.04–1.43]), an increased point estimate but not a significantly increased risk of developing CRC after exposure to GnRH (HR 1.30 [95% CI 0.98–1.72]) or Orchiectomy (HR 1.39 [95% CI 0.95–2.03]), whilst exposure to Total Androgen Blockade (TAB) yielded a decreased point estimate that was not significant (HR 0.87 [95% CI 0.43–1.75]). (Table 2 A). Analysis of latency yielded significantly decreasing HRs over time (p = 0.049 for trend) (Table 3 A).

**Table 2A:** Flexible parametric analysis of hazard ratio (HR) with 95% confidence interval (CI) of colorectal cancer, colon cancer and rectal cancer according to exposure to androgen deprivation therapy (ADT)

	Colorectal cancer				Colon cancer					Rectal cancer				
	No	Yes	HR (95% CI) Crude	HR (95% CI) Adjusted	No	Yes	*	HR (95% CI) Crude	HR (95% CI) Adjusted	No	Yes	*	HR (95% CI) Crude	HR (95% CI) Adjusted
All men	139,303	2,243			140,070	1,447	29			140,750	767	29		
No ADT	113,788	1,810	1.00 (Reference)	Ref.	114,415	1,158	25	Ref.	Ref.	114,946	627	25	Ref.	Ref.
All ADTs	25,515	433	1.31 (1.18–1.45)	1.27 (1.15–1.41)	25,655	289	4	1.37 (1.21–1.56)	1.33 (1.17–1.51)	25,804	140	4	1.21 (1.01–1.45)	1.19 (0.99–1.43)
AA	8,717	139	1.21 (1.02–1.44)	1.23 (1.04–1.47)	8,764	92	0	1.26 (1.02–1.55)	1.29 (1.04–1.60)	8,809	47	0	1.17 (0.87–1.57)	1.19 (0.88–1.60)
GnRH	2,472	51	1.36 (1.03–1.80)	1.30 (0.98–1.72)	2,490	32	1	1.33 (0.94–1.89)	1.29 (0.91–1.83)	2,504	18	1	1.38 (0.86–2.20)	1.27 (0.78–2.06)
GnRH + flare	10,723	174	1.29 (1.10–1.50)	1.22 (1.04–1.43)	10,775	119	3	1.38 (1.14–1.67)	1.29 (1.07–1.56)	10,842	52	3	1.10 (0.83–1.46)	1.07 (0.81–1.42)
ORCH	1,443	28	1.62 (1.12–2.35)	1.39 (0.95–2.03)	1,452	19	0	1.73 (1.10–2.71)	1.42 (0.89–2.27)	1,462	9	0	1.48 (0.77–2.86)	1.38 (0.71–2.66)
TAB	940	8	0.78 (0.39–1.57)	0.87 (0.43–1.75)	944	4	0	0.61 (0.23–1.64)	0.70 (0.26–1.88)	944	4	0	1.12 (0.42–2.99)	1.20 (0.45–3.22)
Unknown	1,220	33	2.19 (1.55–3.10)	2.05 (1.45–2.90)	1,230	23	0	2.40 (1.59–3.63)	2.23 (1.47–3.37)	1,243	10	0	1.87 (1.00-3.50)	1.79 (0.95–3.34)

Flexible parametric model including age, marital status (not married, married, separated and widower) and educational level (low: less than 10 years; intermediate: 10–12 years; high: > 12 years). AA = Anti-Androgens; GnRH = Gonadotropin Releasing Hormone; GnRH + flare = Gonadotropin Releasing Hormone + flare protection; ORCH = Orchiectomy; TAB = Total Androgen Blockade. Due to cases that were diagnosed with both colon and rectal cancer simultaneously and that were included in the analysis of colorectal cancer but excluded from all subgroup analyses these are 29 missing (*) from Colon cancer and Rectal cancer

**Table 3A:** Poisson regression analysis of hazard ratio (HR) with 95% confidence interval (CI) to evaluate a possible latency effect in cancer development for colorectal cancer, colon cancer and rectal cancer in different time periods of exposure to androgen deprivation therapy (ADT)

ADT duration	Colorectal cancer				Colon cancer				Rectal cancer			
	No	Yes	HR (95% CI) Crude	HR (95% CI) Adjusted	No	Yes	HR (95% CI) Crude	HR (95% CI) Adjusted	No	Yes	HR (95% CI) Crude	HR (95% CI) Adjusted
1–3 years	24,965	210	1.37 (1.18–1.59)	1.34 (1.15–1.56)	25,047	128	1.31 (1.08–1.60)	1.28 (1.05–1.56)	25,095	80	1.50 (1.17–1.93)	1.47 (1.14–1.89)
3–5 years	15,589	133	1.30 (1.07–1.56)	1.26 (1.04–1.52)	15,673	98	1.53 (1.22–1.91)	1.47 (1.17–1.84)	15,780	34	0.90 (0.63–1.30)	0.89 (0.62–1.28)
> 5 years	9,234	90	1.06 (0.85–1.33)	1.01 (0.81–1.27)	9,305	63	1.12 (0.86–1.48)	1.05 (0.80–1.38)	9,411	26	0.95 (0.62–1.43)	0.91 (0.60–1.38)
P-value for trend			0.076	0.049			0.441	0.332			0.040	0.034

Poisson regression model including age, marital status (not married, married, separated and widower) and educational level (low: less than 10 years; intermediate: 10–12 years; high: > 12 years). ADT duration (non-cumulative): 1–3; 3–5; >5 years. P value for trend where p < 0.05 is considered significant

### Risk of adenocarcinoma of the colon

During follow-up, 1,447 new cases of adenocarcinoma of the colon were identified. Exposure to any form of ADT among prostate cancer patients was followed by an increased risk of adenocarcinoma of the colon (HR 1.33 [95% CI 1.17–1.51]) as compared to ADT unexposed cancer-free matched controls. Stratified into different forms of ADT, there was a significantly increased risk of developing adenocarcinoma of the colon after exposure to AA (HR 1.29 [95% CI 1.04–1.60]) and GnRH + flare (HR 1.29 [95% CI 1.07–1.56]), but no significant association after exposure to GnRH (HR 1.29 [95% CI 0.91–1.83]), Orchiectomy (HR 1.42 [95% CI 0.89–2.27]), or Total Androgen Blockade (TAB) (HR 0.70 [95% CI 0.26–1.88]), (Table 2 A). Analysis of latency yielded no significant time trend (p = 0.332) (Table 3 A). Among 1,447 cases of adenocarcinoma of the colon identified during follow up, 738 were located in the proximal colon. Exposure to ADT among prostate cancer patients was not associated with a significantly increased risk of developing adenocarcinoma of the proximal colon (any ADT HR 1.13 [95% CI 0.93–1.36]) (Table 2B) and analysis of latency yielded no significant time trend (p = 0.363) (Table 3B). Among 1,447 cases of adenocarcinoma of the colon identified during follow up, 636 were located in the distal colon. Exposure to any form of ADT among prostate cancer patients was followed by an increased risk of adenocarcinoma of the distal colon (HR 1.53 [95% CI 1.26–1.85]), particularly after exposure to GnRH + flare (HR 1.59 [95% CI 1.21–2.08]) (Table 2B). Analysis of latency yielded inconclusive results with an initially increased HR followed by a decreased HR (p = 0.723 for trend) (Table 3B).

**Table 2B:** Flexible parametric analysis of hazard ratio (HR) with 95% confidence interval (CI) of proximal colon cancer and distal colon cancer according to exposure to androgen deprivation therapy (ADT)

	Proximal colon cancer				Distal colon cancer			
	No	Yes	HR (95% CI) Crude	HR (95% CI) Adjusted	No	Yes	HR (95% CI) Crude	HR (95% CI) Adjusted
All men	140,070	738			140,070	636		
No ADT	114,415	610	1.00 (Reference)	Ref.	114,415	493	Ref.	Ref.
All ADTs	25,655	128	1.16 (0.96–1.40)	1.13 (0.93–1.36)	25,655	143	1.58 (1.31–1.91)	1.53 (1.26–1.85)
AA	8,764	43	1.12 (0.82–1.52)	1.18 (0.86–1.61)	8,764	43	1.37 (1.00-1.87)	1.37 (1.00-1.88)
GnRH	2,490	15	1.19 (0.71–1.98)	1.14 (0.68–1.90)	2,490	14	1.37 (0.80–2.32)	1.33 (0.78–2.26)
GnRH + flare	10,775	51	1.13 (0.85–1.50)	1.05 (0.79–1.39)	10,775	62	1.68 (1.29–2.19)	1.59 (1.21–2.08)
ORCH	1,452	9	1.56 (0.81–3.01)	1.29 (0.67–2.48)	1,452	10	2.12 (1.13–3.96)	1.76 (0.90–3.41)
TAB	944	0	-	-	944	4	1.43 (0.53–3.82)	1.54 (0.58–4.14)
Unknown	1,230	10	2.00 (1.07–3.73)	1.79 (0.96–3.35)	1,230	10	2.42 (1.29–4.54)	2.34 (1.25–4.38)

Flexible parametric model including age, marital status (not married, married, separated and widower) and educational level (low: less than 10 years; intermediate: 10–12 years; high: > 12 years). AA = Anti-Androgens; GnRH = Gonadotropin Releasing Hormone; GnRH + flare = Gonadotropin Releasing Hormone + flare protection; ORCH = Orchiectomy; TAB = Total Androgen Blockade. Due to ICD-7: 153.9 (overlapping lesion of the colon; colon unspecified including large intestine NOS) that was included in the analysis of colorectal and colon cancer but excluded from the sub-site analyses of proximal and distal colon cancer there are 73 missing (1447-738-636 = 73) cases of colon cancer from this table

**Table 3B:** Poisson regression analysis of hazard ratio (HR) with 95% confidence interval (CI) to evaluate a possible latency effect in cancer development for proximal colon cancer and distal colon cancer in different time periods of exposure to androgen deprivation therapy (ADT)

ADT duration	Proximal colon cancer				Distal colon cancer			
	No	Yes	HR (95% CI) Crude	HR (95% CI) Adjusted	No	Yes	HR (95% CI) Crude	HR (95% CI) Adjusted
1–3 years	25,119	56	1.13 (0.85–1.51)	1.11 (0.83–1.49)	25,114	61	1.38 (1.04–1.83)	1.33 (1.00-1.77)
3–5 years	15,727	44	1.25 (0.90–1.74)	1.21 (0.87–1.68)	15,722	49	1.88 (1.36–2.61)	1.81 (1.30–2.52)
> 5 years	9,340	28	0.94 (0.63–1.40)	0.86 (0.58–1.29)	9,335	33	1.45 (0.99–2.11)	1.39 (0.95–2.03)
P-value for trend			0.514	0.363			0.714	0.723

Poisson regression model including age, marital status (not married, married, separated and widower) and educational level (low: less than 10 years; intermediate: 10–12 years; high: > 12 years). ADT duration (non-cumulative): 1–3; 3–5; >5 years. P value for trend where p < 0.05 is considered significant

### Risk of rectal adenocarcinoma

During follow-up, 767 cases of rectal adenocarcinoma were identified. There was no significant increased risk of developing rectal adenocarcinoma among prostate cancer patients exposed to either any form of ADT (HR 1.19 [95% CI 0.99–1.43]) or different forms of ADT (Table 2 A). Examination of latency effects showed an initially increased HR followed by decreased point estimates which taken together yielded significantly decreased HRs over time (p = 0.034 for trend) (Table 3 A).

## Discussion

The results of this study indicate that prostate cancer patients exposed to ADT were at an increased risk of CRC, in particular of the distal colon as compared to ADT unexposed cancer-free matched controls. Furthermore, stratified into different forms of ADT, the increased risk was especially evident after exposure to both AA and GnRH + flare. However, time trends indicated that this increased risk was attenuated by time which argues against a dose-response effect.

The strengths of this study lie in the availability of nationwide, comprehensive, and high-quality health-care registers, yielding a large sample size with a substantial number of person-years at risk, a close to complete follow-up and a documented accuracy of cancer diagnoses through ascertainment with the national cancer register. All these factors serve to counteract selection and information biases. As was the case in earlier studies [38, 39], but as an evolution compared to recent findings [18], this study made use of data on the type of ADT prescribed to each prostate cancer patient. Moreover, the number of CRCs classified as unspecified site was small providing robust risk estimates in the overall sub-site analysis and all proximal and distal portions of the colon were evaluated separately.

A number of weaknesses should be highlighted. Follow-up was shorter than we would have liked, despite prostate cancer data being available in the NPCR since 1998, due to the relatively short time since the inception of the Prescribed Drug Register in Sweden in 2005 and the latest version of PCBaSe (version 4) only covering cases through 2016. However, we don’t consider follow-up too short to capture a biologically relevant duration of exposure. One reason for this is that in previous research [39] that reported a dose-response trend of increased CRC risk with a longer duration of ADT exposure, this effect appeared to arise relatively early, perhaps within one year, suggesting that ADT might influence relatively late processes of carcinogenesis. Despite a large sample size, we may have lacked the statistical power in secondary analyses. The stratified analyses of different forms of ADT were hampered by a small number of cases in certain subgroups, especially with regard to proximal and distal colon cancer, resulting in wider CIs and, therefore, less precise estimates. Moreover, our main finding of an increased risk of colorectal cancer could be due to a type I error i.e., a chance finding. However, our main hypothesis of an increased risk of CRC was formulated à priori and based on previously published results. Our results could have been impacted by detection or surveillance bias i.e., an increase in a second primary cancer (CRC) following a diagnosis of a primary cancer (prostate cancer). We tried to reduce the impact of such bias by using a latency time of one year.

Another limitation to our study is the lack of data on a number of potential confounders known to be associated with CRC which might also influence the use of ADT: obesity, diabetes, diet, and other lifestyle-related risk factors [38]. Adverse effects of ADT include the development of known risk factors for CRC such as obesity and diabetes, neither of which were included in our database. One study [39] stated that obesity may be an important confounder because it is known to increase the risk of CRC in men and is possibly associated with advanced prostate cancer which is more likely to be treated with ADT. Another potential concern is that we did not include prostate cancer stage in our analysis. It is conceivable that our study population with a mean age of approximately 75 years and exposed to ADT are more likely to have advanced prostate cancer. Due to both prostate cancer per se and potential side effects of ADT including obesity these patients might be more physically inactive, which has been associated with an increased risk of CRC [40]. Finally, regarding factors such as diet and other lifestyle-related risk factors not included in our database, two studies [18, 38] found that it was unlikely that these factors could have affected the validity of their results as it is unclear why these variables would be differentially distributed between users and non-users of ADT.

Despite all of these potential confounders, other important variables such as age, residential area, marital status, and educational level (a robust measure of socioeconomic status) were adjusted for in the model.

A further limitation to this study which might introduce biases related to misclassification of exposure during follow-up, is that we only had information on the first prescription of ADT. Hence, we assumed prostate cancer cases to remain exposed to the same type of ADT until the end of follow-up. The same assumption that was used in a previous study [39]. Furthermore, we treated prostate cancer patients exposed to ADT as a time-constant exposure and assumed, based on previous research, that prostate cancer patients have a high adherence to treatment [41] although age above 75 years and low-risk prostate cancer were associated with lower adherence. Even so, it was unknown to us whether prescriptions were actually filled at the pharmacy or to what extent patients fully complied with the treatment regimen. However, all such misclassification of exposure resulting mostly from non-adherence to prescribed treatment in an aged population would only serve to dilute the point estimates toward the null.

We opted to only use a control group from the general population as opposed to or including a control group of prostate cancer patients that were not exposed to ADT for several reasons: Firstly, men diagnosed with prostate cancer that do not receive ADT will very likely differ regarding several important potentially controllable but also uncontrollable factors from those treated with ADT which could introduce bias. Secondly, men diagnosed with prostate cancer that do not receive ADT are in fact a heterogenous group considering that many of them have a low-risk prostate cancer that never requires any treatment, while others have tumors that are of higher risk treated with radiotherapy or surgery. Both of these examples of differences within the prostate cancer group raise concerns of bias, particularly confounding by indication which might have uncontrollable and unpredictable effects on the results. Moreover, many prostate cancer patients will experience disease progression and will eventually receive ADT as this is used for palliation for advanced disease. Using men diagnosed with prostate cancer not receiving ADT as a control group would therefore risk adding complexity since many will become exposed which would also raise questions about its robustness and appropriateness as comparison group. Lastly, confining the study population only to men diagnosed with prostate cancer would raise concerns on its external validity and relevance for the general population, i.e., the aim of the study.

This study found an increased risk of CRC, in particular an increased risk of adenocarcinoma of the distal colon among prostate cancer patients exposed to ADT as compared to ADT unexposed cancer-free matched controls. A diagnosis of prostate cancer may lead to a positive change in lifestyle, potentially influencing the exposure to risk factors such as diet, tobacco smoking and obesity, lowering the risk of further malignancy, including CRC. However, such a change in lifestyle would only serve to weaken the association, not refute it. Furthermore, it is conceivable that men with prostate cancer may be more susceptible to the development of CRC. A lower risk of CRC after prostate cancer was reported in one study [42] whereas others reported an increased risk of CRC after a diagnosis of prostate cancer [43, 44].

The association between prostate cancer patients exposed to ADT and an increased risk of CRC is in support with some similar studies [18, 39] but in conflict with others [38]. Gillessen et al. [39] reported the highest risk of CRC after orchiectomy followed by exposure to GnRH. These associations remained after adjusting for potential confounders such as obesity and diabetes. They also found a dose-response effect with a higher risk of CRC with increasing duration of ADT possibly supporting a causal association, whereas our analysis of latency yielded a negative dose-response trend in CRC with significantly decreasing HRs following a longer duration of ADT, weakening the potential for a causal association between prostate cancer patients exposed to ADT and CRC. However, Gillessen’s study was performed entirely among men with prostate cancer potentially introducing bias from confounding by indication. Lu et al. similarly found an increased risk of CRC after orchiectomy as well as an increased risk following ADT. The increased risk was found in the distal colon, which is in line with our results, but also in the rectum [18]. In contrast to these positive studies Assayag et al. similarly found an increased risk of CRC after orchiectomy but not a significantly increased risk following exposure to ADT and no dose-response effect. They hypothesized that it is unlikely that the anti-hormonal effects of GnRH agonists and orchiectomy act differently on colorectal tissues and that the association observed between bilateral orchiectomy and colorectal cancer is better explained by the longer anti-hormonal duration of castration [38]. Since we did not observe any significant effect following orchiectomy on overall CRC risk or after sub-site analysis, we cannot confirm their hypothesis, possibly due to small numbers. Others have found an increased risk of CRC following a diagnosis of prostate cancer but did not include ADT as a separate exposure variable [45]. Two separate studies further concluded that one should not assume that an increased rate of a second cancer is necessarily linked to the treatment of the first cancer [46] and that treatment might not be the only contributor to a higher risk of colon cancer suggesting that similarities in etiologic factors also could contribute [47].

We hypothesized that the risk of CRC following exposure to ADT in prostate cancer patients would be sub-site dependent since the proximal and distal colorectum differ in genetic pathways to disease, the influence of sex-related factors and the distribution of sex hormone receptors [18]. Furthermore, the incidence of CRC in men has consistently been shown to be higher in the distal part of the colon and rectum compared to females [48]. In our all-male cohort, we did indeed find an increased risk in the distal part of the colon.

Endogenous sex hormones and CRC risk has been studied extensively in both experimental and epidemiological settings, but their role remain unclear. Previous research has indicated sex-specific effects of testosterone on CRC development. Higher endogenous testosterone levels was associated with a lower risk of CRC in men [15] but a higher risk of CRC [16] and advanced adenoma [49] in postmenopausal women. However, these results have not been consistently reproduced in the literature and several studies [50–52] have found no association between testosterone and CRC risk in both men and women. A recent review and meta-analysis [53] found no associations between endogenous sex steroid hormones in men or postmenopausal women and CRC risk with substantial heterogeneity among women.

The biological mechanisms behind the potentially true association between prostate cancer patients exposed to ADT and CRC risk remain unclear. Androgen receptors are more frequently found in normal colonic mucosa than in colorectal tumors [54, 55]. Several animal studies suggest that androgens may have a protective effect on the development of CRC whereas suppression of androgens may promote it [56–58]. In spite of these proposed molecular mechanisms for androgens acting through androgen receptors and their role in CRC carcinogenesis most previous epidemiological and experimental studies show opposing results. There seems to be a relationship between androgens (and therefore ADT) and the development of CRC but this relationship seems to be far more complex and involve both direct mechanisms (androgen receptors) and indirect mechanisms such as stress hormone levels, the innate immune system, bile as well as various risk factors including type 2 diabetes mellitus and metabolic syndrome or a combination of both acting in synergy [59]. These mentioned strong risk factors for CRC are correlated to hyperinsulinemia [60], which together with insulin resistance are linked to obesity and CRC development [61]. Since ADT can cause obesity and hyperinsulinemia which in turn increases the risk of CRC [62] it is conceivable that insulin resistance as a consequence of ADT is another plausible mechanism for the increased CRC risk. Moreover, both androgens and estrogens are hypothesized to prevent tumor growth in part by preventing insulin and insulin-like growth factor (IGF) from binding to their receptors [63] and there is evidence of an association between IGF-1 and the risk of CRC [64]. Furthermore, men with prostate cancer who received endocrine treatment and underwent surveillance were at greater risk of developing IGF-1-related cancers [47]. Thus, obesity as well as metabolic syndrome are risk factors for both prostate cancer [65] as well as CRC, and both are linked to high IGF-1 levels, which in turn is associated with an increased risk of both prostate cancer and CRC [64, 66]. It is clear that future studies should include detailed information on obesity which was not included in our database. An increased risk of colon but not rectal cancer in men, corroborating our findings, was found in one study [67] with increasing anthropometric measurements of obesity alone. One should further take into consideration that although obesity has also been associated with an increased risk of CRC in women, the association seems to be stronger in men and only in men is obesity associated with lower testosterone levels [68]. Increased levels of endogenous estrogen seem to protect women whereas increased levels of endogenous testosterone might decrease the risk of CRC in men [15]. Since increasing adiposity with age leads to higher levels of estrogen in postmenopausal women, whereas it leads to a decline in testosterone levels in men, the relationship between obesity and CRC risk might be attenuated in women but amplified in men [11].

During the past decades, research has failed to reach a consensus regarding the direct connection between androgens and the risk of CRC. Taken together, all previously mentioned epidemiological and experimental evidence to date infer the possibility that the findings in this study may not be causal but a result of an interplay between true sex hormonal effects, the possibility that men with prostate cancer are at greater risk of developing CRC à priori compared to the general population, the impact of known side effects of ADT such as obesity, hyperinsulinemia and insulin resistance which are known risk factors for CRC and the added effects of various forms of residual confounding. Thus, further research is needed that will focus on both epidemiologic data and the molecular mechanisms implicated in CRC, to provide the answer to the role of androgens, and therefore the effect of anti-androgens, in the etiology of CRC.

If the results of the present study can be further verified, the potential future clinical implications may include intensified screening programs for CRC in prostate cancer patients exposed to ADT and a continued awareness that the potential long-term effects of ADT constitute potential risk factors for the development of CRC. However, further research to clarify these relationships is warranted before any large preventive measures are instituted in these populations. Moreover, research should focus on elucidating the biological mechanisms behind possible direct effects of androgens and their receptors in the etiology of CRC to potentially harness any preventive effects and future treatment of prostate cancer patients should strive to employ different forms of ADT with less side effects contributing to an increased risk of CRC.

In conclusion, this Swedish nationwide population-based matched cohort study yielded an increased risk of CRC and more specifically adenocarcinoma of the distal colon, suggesting an increased association between androgen deprivation therapy in prostate cancer patients and CRC. However, we found no dose-response effect which argues against a true causal effect. Future studies are warranted.

## Data Availability

All data and materials are available upon request.

## Code Availability

All statistical analyses were performed using the statistical software Stata (version 16; Stata Corporation, College Station, TX, USA). All coding is available upon request.
